# miR-100-5p and miR-203a-3p suppress esophageal squamous cell carcinoma progression by targeting FKBP5

**DOI:** 10.3892/or.2025.9003

**Published:** 2025-10-03

**Authors:** Hiroto Tanaka, Suguru Maruyama, Katsutoshi Shoda, Yoshihiko Kawaguchi, Yudai Higuchi, Takaomi Ozawa, Takashi Nakayama, Ryo Saito, Wataru Izumo, Koichi Takiguchi, Kensuke Shiraishi, Shinji Furuya, Hidetake Amemiya, Hiromichi Kawaida, Daisuke Ichikawa

**Affiliations:** Department of Digestive Surgery, University of Yamanashi, Chuo, Yamanashi 409-3898, Japan

**Keywords:** esophageal cancer, ESCC, poor differentiation, FKBP5, miR-100-5p, miR-203a-3p

## Abstract

Poorly differentiated cancers, including esophageal squamous cell carcinoma (ESCC), exhibit higher malignant potential and worse prognoses than well-differentiated types. The present study aimed to identify microRNAs (miRNAs or miRs) involved in ESCC progression and their target mRNAs, focusing on tumor differentiation. miRNA candidates were selected using a miRNA array-based approach and GEO datasets, comparing expression levels between poorly and non-poorly differentiated ESCC. Clinical samples (n=61) and cell lines were analyzed to determine the significance and function of the selected miRNAs and their target mRNA. miR-100-5p and miR-203a-3p were significantly downregulated in poorly differentiated ESCC, with lower expression strongly associated with poorer overall survival (OS) (miR-100-5p: P=0.02; miR-203a-3p: P=0.05) and relapse-free survival (RFS) (miR-100-5p: P=0.04; miR-203a-3p: P=0.12). Overexpression of these miRNAs suppressed cell migration and invasion. *FKBP5* was identified as a common target, with its expression significantly reduced upon double-transfection with miR-100-5p and miR-203a-3p. *FKBP5* downregulation reduced tumor aggressiveness in KYSE70 cells, and clinical samples showed significantly worse survival rates in patients with high *FKBP5* expression (OS: P=0.02; RFS: P=0.04). These findings suggest that miR-100-5p and miR-203a-3p act as tumor suppressors by targeting *FKBP5*, highlighting *FKBP5* as a potential therapeutic target in ESCC.

## Introduction

Esophageal cancer is one of the most common solid tumors and the seventh leading cause of cancer-related death worldwide (1). Despite advancements in treatment approaches, the prognosis for patients with advanced esophageal cancer remains poor (2). The most common histologic subtypes of esophageal cancer are esophageal squamous cell carcinoma (ESCC) and esophageal adenocarcinoma. In Asia, ESCC accounts for a particularly high proportion of cases.

Regarding ESCC, differentiation grades are classified into three categories: Well-differentiated, moderately differentiated, and poorly differentiated, according to the Union for International Cancer Control (UICC) Staging Manual, 8th edition. Well-differentiated tumors are characterized by prominent keratinization, including pearl formation, along with a minor component of non-keratinizing basal-like cells. Moderately differentiated tumors exhibit a range of histological features, from parakeratotic changes to poorly keratinizing lesions. Poorly differentiated tumors mainly consist of basal-like cells forming both large and small nests, often accompanied by central necrosis. Furthermore, these nests can form sheets or pavement-like arrangements of tumor cells and may occasionally include small numbers of parakeratotic or keratinizing cells (3). Poorly differentiated ESCC typically has a greater propensity for early lymphatic metastasis and skip metastasis to distant lymph node stations, leading to a poorer prognosis (4,5).

Meanwhile, microRNAs (miRNAs or miRs) have gained attention for their oncogenic or tumor-suppressive functions. These endogenous, small, non-coding, single-stranded RNAs, measuring 20–25 nucleotides, regulate target gene expression at the post-transcriptional level by binding to complementary sequences (6,7). Therefore, it was hypothesized that specific miRNAs and their target mRNAs play crucial roles in ESCC.

In the present study, it was aimed to identify miRNAs associated with tumor characteristics in ESCC by comparing miRNA expression between poorly differentiated (por) and well- to moderately differentiated (non-por) ESCC using a comprehensive miRNA array-based approach with clinical tissue samples and GEO datasets from the National Library of Medicine. It was also aimed to identify mRNA targeted by specific miRNAs to better understand the mechanisms of tumor progression and potential therapeutic targets in ESCC.

## Materials and methods

### Microarray and bioinformatics

To identify potential biomarkers, microarray analyses of ESCC tissue samples were first performed using 3D-Gene miRNA oligo chips (Toray Industries, Inc.), each containing 2,565 genes. miRNA expression in tumor tissues was collected from patients with ESCC who underwent curative resection at our institution, comparing miRNA expression between non-por and por ESCC within the same specimens. RNAs were labeled using the 3D-Gene miRNA labeling kit (Toray Industries, Inc.). Fluorescent signals were scanned with a 3D-Gene scanner 3000 (Toray Industries, Inc.) and analyzed using the 3D-Gene Extraction software (Toray Industries, Inc.). Next, our analysis was supplemented with a GEO dataset (GSE43732) (8), a publicly accessible repository that includes miRNA expression profiles from primary lesions of 119 patients with ESCC. miRNA expression was compared between patients with por and non-por ESCC and additionally performed overall survival (OS) analysis according to tumor differentiation status.

### Patients and samples

The study population comprised a retrospective cohort of patients with consecutive ESCC who underwent esophagectomy at the Department of Digestive Surgery, University of Yamanashi (Chuo, Japan) between February 2008 and January 2023. To eliminate any confounding effects of chemotherapy or radiotherapy, 169 patients who received neoadjuvant chemotherapy (n=144) or neoadjuvant chemoradiotherapy (n=25) were excluded. An additional 11 cases were excluded due to non-curative resection. Furthermore, 30 cases unsuitable for RNA extraction were removed because the tissue samples were too shallow for adequate RNA retrieval. As a result, a final cohort of 61 patients with ESCC were eligible for the present study (Fig. S1). Clinicopathological characteristics of patients (including sex and age distribution) are listed in Table I.

Tumor specimens and resected lymph nodes obtained at the time of surgery were immediately fixed in 10% neutral-buffered formalin at room temperature for 24 h and embedded in paraffin after fixation (FFPE). The clinical and pathological tumor stages of esophageal cancer were classified according to the UICC TNM staging, 8th Edition (3).

Patients were followed up with physical examinations, blood tests, and computed tomography every 4 months for at least 1 year, and every 6 months thereafter. The present study was approved (approval no. 2723) by the Ethics Committee of Yamanashi University (Chuo, Japan) and conducted in accordance with the ethical standards of the Declaration of Helsinki and its amendments.

### Histopathological evaluation and immunohistochemical (IHC) staining analyses

Serial sections were obtained from FFPE tissue blocks for IHC staining and RNA extraction. FFPE sections (5 µm) were used for IHC. After deparaffinization with xylene, dehydration using a graded ethanol series, and antigen retrieval at 120°C for 15 min endogenous peroxidase activity was inhibited (Peroxidase-Blocking Solution; cat. no. S2023; Dako; Agilent Technologies, Inc.), and non-specific binding was blocked (Protein Block Serum-Free; cat. no. X0909; Dako; Agilent Technologies, Inc). The sections were then incubated with a polyclonal *FKBP5* antibody (1:50; cat. no. 14155-1; Proteintech Group, Inc.) at room temperature for 1.5 h. Subsequently, slides were treated with an anti-rabbit secondary antibody (EnVision^+^ HRP; cat. no. K4003; Dako; Agilent Technologies, Inc.) at room temperature for 60 min. DAB working solution was used to stain positive cells. The results were observed and recorded using optical microscopy (BZ-X810; Keyence Corporation).

Each slide was scored according to the H-score, which is calculated by multiplying two parameters: the proportion of positive cells (1–100%) and the intensity of staining (1=weak; 2=moderate; 3=strong; range, 0–300).

### Western blot analysis

Total proteins were obtained from the harvested KYSE70 cells, and protein concentrations were determined using the bicinchoninic acid (BCA) protein assay kit (Thermo Fisher Scientific, Inc.). Cell lysate proteins (20 µg/lane) were separated by 10% sodium dodecyl sulfate-polyacrylamide gel electrophoresis (SDS-PAGE) and then transferred onto polyvinylidene fluoride membranes. After blocking with StartingBlock Blocking Buffer (Thermo Fisher Scientific, Inc.) at room temperature for 1 h, the membranes were incubated overnight at 4°C with the following antibodies: E-cadherin (1:1,000; cat. no. 20874-1-AP; Proteintech Group, Inc.), Vimentin (1:2,000; cat. no. 10366-1-AP; Proteintech Group, Inc.), phosphorylated (p-)PI3K (1:1,000; cat. no. 17366; Cell Signaling Technology, Inc.), PI3K (1:1,000; cat. no. 11889; Cell Signaling Technology, Inc.), p-AKT (1:1,000; cat. no. 4060; Cell Signaling Technology, Inc.), AKT (1:1,000; cat. no. 4691; Cell Signaling Technology, Inc.), GAPDH (1:50,000; cat. no. 60004-1-lg; Proteintech Group, Inc.). Then, the membranes were incubated with goat anti-rabbit or anti-mouse IgG conjugated to horseradish peroxidase (1:10,000; cat. nos. ab6721 and ab6789; Abcam) buffer for 1 h at room temperature. The membranes then were probed with the indicated antibodies, and proteins were detected by a western blot HRP Substrate (Takara Bio, Inc.). Densitometric analysis of the protein bands was performed using FIJI (ImageJ; version 1.54q, National Institutes of Health).

### Reverse transcription-quantitative polymerase chain reaction (RT-qPCR)

FFPE tissue samples were cut into 10-µm-thick sections, and total RNA was extracted using the miRNeasy FFPE kit (Qiagen GmbH) and NucleoSpin miRNA (Takara Bio, Inc.), following the manufacturer's protocols. A NanoDrop 2000 spectrophotometer (Thermo Fisher Scientific, Inc.) was used to measure the total RNA concentration.

Reverse transcription was performed using the High-Capacity cDNA Reverse Transcription Kit (Thermo Fisher Scientific, Inc.) and the TaqMan MicroRNA Reverse Transcription Kit (Thermo Fisher Scientific, Inc.). Total RNA and miRNA levels were quantified by RT-qPCR following standard procedures.

RT-qPCR conditions were as follows: Initial denaturation at 95°C for 10 min, followed by 40 cycles of 95°C for 15 sec and 60°C for 60 sec. Total RNA levels were normalized to the endogenous control gene glyceraldehyde-3-phosphate dehydrogenase (GAPDH), while miRNA levels were normalized to RNU6B.

The following primers were used for the TaqMan assay (Thermo Fisher Scientific, Inc.): human hsa-miR-100-5p (cat. no. 78224_mir; 5′-AACCCGUAGAUCCGAACUUGUG-3′), hsa-miR-203a-3p (cat. no. 478316_mir; 5′-GUGAAAUGUUUAGGACCACUAG-3′), *FKBP5* (cat. no. Hs01561006_m1), GAPDH (cat. no. Hs02786624_g1) and RNU6B (cat. no. 001093; 5′-CGCAAGGATGACACGCAAATTCGTGAAGCGTTCCATATTTTT-3′). ΔCq values for all RNAs were calculated relative to the control genes GAPDH and RNU6B. Relative mRNA and miRNA expression levels were evaluated using the 2^−ΔΔCq^ method (9).

### Cell culture

The poorly differentiated ESCC cell lines [KYSE70 (JCRB0190), KYSE110 (JCRB1064), and TE5 (RCB1949)] and the well- to moderately differentiated ESCC cell lines [TE4 (RCB2097), TE8 (RCB2098), and TE11 (RCB2100)] were used in the present study. KYSE70 and KYSE110 were obtained from the Japanese Collection of Research Bioresources (JCRB) Cell Bank, while TE series cell lines were obtained from the RIKEN BRC Cell Bank in 2024. All cell lines were confirmed to be free of mycoplasma contamination, as previously described (10–12).

KYSE70, TE4, TE8 and TE11 were cultured in Roswell Park Memorial Institute (RPMI)-1640 medium (Nacalai Tesque, Inc.) supplemented with 10% fetal bovine serum (FBS) and 1% penicillin-streptomycin (Sigma-Aldrich; Merck KGaA). The other ESCC cell lines were cultured in Ham's F-12 medium (FUJIFILM Wako Pure Chemical Corporation) supplemented with 2% FBS and penicillin-streptomycin (Sigma-Aldrich, Merck KGaA). All cells were maintained in a humidified incubator at 37°C with 5% carbon dioxide.

### Transfection of KYSE70 with miRNA mimics

The miRNA mimics for miR-100-5p (mirVana miRNA mimic; cat. no. MC10188; 5′-AACCCGUAGAUCCGAACUUGUG-3′), miR-203a-3p (mirVana miRNA mimic; cat. no. MC10152; 5′-GUGAAAUGUUUAGGACCACUAG-3′, and the miRNA mimic negative control (mirVana miRNA Mimic Negative Control #1; cat. no. 4464058) were purchased from Thermo Fisher Scientific, Inc. Transfections were performed using Lipofectamine 2000 (Thermo Fisher Scientific, Inc.) according to the manufacturer's protocol.

The final concentration for *miR-100-5p* and *miR-203a-3p* mimics, as well as the negative control, was 10 nM. For double transfection, both mimics were used at 10 nM each. Total RNA and protein were collected 48 h post-transfection. Transfection efficiency was assessed by quantifying miRNAs expression levels using RT-qPCR.

### Transfection of KYSE70 with siRNA

The siRNA for *FKBP5* (Silencer Select siRNA; cat. no. s5215) (sense sequence: GAGAAAGGCUUGUAUAGGAtt) and the siRNA negative control (Silencer Select Negative Control #1; cat. no. 4390843) were purchased from Thermo Fisher Scientific, Inc. The transfection protocol and concentration were the same as those used for the mimics. Knockdown efficiency of *FKBP5* was confirmed by western blotting.

### Migration and invasion assays

The cell migration and invasion assays for KYSE70 cells were conducted using Transwell chambers. For invasion assays, the chambers were pre-coated with Matrigel (Corning, Inc.), while migration assays were performed without it. Cells (1×10^5^) suspended in serum-free medium were placed in the upper chamber, while 10% FBS was added to the lower chamber. After a 24-h incubation, cells were stained using the Differential Quik III Stain Kit (Polysciences, Inc.). Following washing and drying, images were captured and examined under the microscope.

### Functional rescue experiments

To determine whether the effects of the double-transfection of miR-100-5p and miR-203a-3p mimics on cell migration and invasion were mediated through *FKBP5* suppression, functional rescue experiments were performed by co-transfecting the double-transfection condition with *FKBP5* siRNA. KYSE70 cells were transfected with the double-transfection of miR-100-5p and miR-203a-3p mimics (10 nM each), with or without *FKBP5* siRNA (10 nM). After 48 h, migration and invasion assays were conducted as aforementioned. The number of migrated and invaded cells in the double-transfection + *FKBP5* siRNA group was compared with that in the double-transfection group to assess whether additional *FKBP5* knockdown further modified the miRNA-induced phenotype.

### Cytotoxicity assay

To assess cell proliferation, KYSE70 cells (1×10^4^/well) were passaged in 96-well plates in RPMI-1640 culture medium containing penicillin-streptomycin, supplemented with 10% FBS. Cell proliferation was measured by the OD values using Cell Counting Kit-8 (CCK-8; (Dojindo Laboratories, Inc.). A total of 100 µl of CCK-8 solution was added to each well, and the plates were incubated for 2 h at 37°C in a CO_2_ incubator. The absorbance was measured at 450 nm on a microplate reader (Spectra Max ABS Plus, Molecular Devices, LLC).

### Apoptosis assays

Apoptosis was assessed in KYSE70 cells following *FKBP5* knockdown. The siRNA transfection procedure and concentrations were identical to those aformentioned. A total of 48 h after transfection, apoptotic activity was evaluated by both flow cytometry and western blotting.

For flow cytometric analysis, cells were collected, washed twice with cold PBS, and resuspended in binding buffer. Annexin V-FITC and propidium iodide (PI) (Nacalai Tesque, Inc.) were added according to the manufacturer's instructions, and samples were analyzed using a flow cytometer (BD FACSCelesta; BD Biosciences) with BD FACSDiva software (v8.0.1.1; BD Biosciences). The proportions of viable, early apoptotic, and late apoptotic cells were quantified.

For western blotting, protein expression of apoptosis-related markers was examined using antibodies against Caspase-3 (1:1,000; cat. no. 14220; Cell Signalling Technology, Inc.) and Bcl-2 (1:1,000; cat. no. ab692; Abcam).

### Bioinformatics analyses

RNA-seq transcriptome data of ESCC was obtained from The Cancer Genome Atlas database (https://portal.gdc.cancer.gov/). Potential miRNA target genes were predicted using TargetScan Human 8.0 (https://www.targetscan.org/vert_80/). To investigate the biological functions of *FKBP5*, Pearson's correlation coefficients were first calculated between *FKBP5* expression and all other genes across ESCC samples. Genes with |Pearson r|>0.3 and P<0.001 were considered significantly correlated with *FKBP5* and subjected to enrichment analysis. Gene Ontology (GO) Biological Process and Kyoto Encyclopedia of Genes and Genomes (KEGG) pathway enrichment were performed using the R package clusterProfiler (version 4.8; http://bioconductor.org/packages/clusterProfiler), with multiple-testing correction by Benjamini-Hochberg and significance thresholds of adjusted P<0.05 and q-value <0.05.

To assess pathway-level associations, single-sample Gene Set Enrichment Analysis (GSEA) was applied using the GSVA package (version 1.42; http://bioconductor.org/packages/GSVA). Each tumor received a score for the following two gene sets from MSigDB: HALLMARK_EPITHELIAL_MESENCHYMAL_TRANSITION, KEGG_PI3K_AKT_SIGNALING_PATHWAY (hsa04151).

Finally, pre-ranked GSEA was conducted with cluster Profiler: GSEA by ranking all genes in descending order of Pearson's correlation with *FKBP5*. Only the EMT (Hallmark) and PI3K-Akt (KEGG) gene sets were evaluated for enrichment, using an FDR cutoff of 0.25. All analyses were performed in R (version 4.4.0; R Foundation for Statistical Computing).

### Statistical analysis

Statistical analysis was performed using GraphPad Prism version 10 (GraphPad Software, Inc.; Dotmatics). Unless otherwise stated, quantitative values were expressed as the mean ± standard deviation (SD). Statistical significance was assessed using the Mann-Whitney U test, Fisher's exact test, and one-way analysis of variance (ANOVA) for each time point, followed by Tukey's post hoc test. OS was calculated from the date of surgery to either death or the last follow-up. Relapse-free survival (RFS) was measured from the date of surgery to recurrence, death, or the last follow-up. Survival curves were generated using the Kaplan-Meier method and compared using the log-rank test. P<0.05 was considered to indicate a statistically significant difference.

## Results

### Identification of miRNA candidates

**Figure 1.**
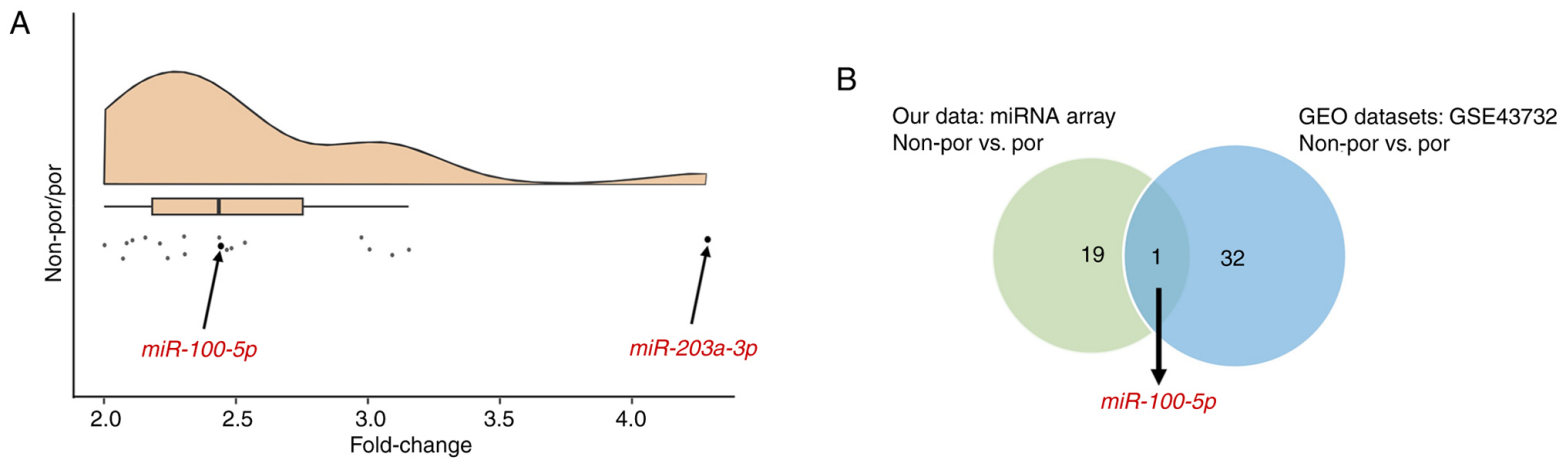
(A) miRNA expression ratio in non-por portion relative to por portion of the tumor using global normalization. (B) Venn diagram showing miRNAs more highly expressed in non-por than por in both microarray and GEO datasets. miRNA or miR, microRNA; por, poorly differentiated; non-por, well- to moderately differentiated.

First, miRNA candidates were identified using a miRNA array-based approach in combination with the GEO datasets. The expression levels of each miRNA were compared between por and non-por ESCC. miR-203a-3p was selected, which exhibited a four-fold difference in expression during microarray analysis, ensuring that the signal intensity of each miRNA was adequate (Fig. 1A). Additionally, miR-100-5p was selected, which exhibited a two-fold difference in expression during microarray analysis, supported by the GEO datasets (Fig. 1A and B).

Furthermore, analysis of a publicly available GEO dataset revealed a consistent trend toward poorer prognosis in patients with por ESCC (Fig. S2). Although this difference was not statistically significant, por ESCC is generally considered more aggressive, and this trend may have biological relevance. Consequently, miR-100-5p and miR-203a-3p were chosen for further analyses in the present study.

### Clinical significance of miR-100-5p and 203a-3p in patients with ESCC

**Figure 2.**
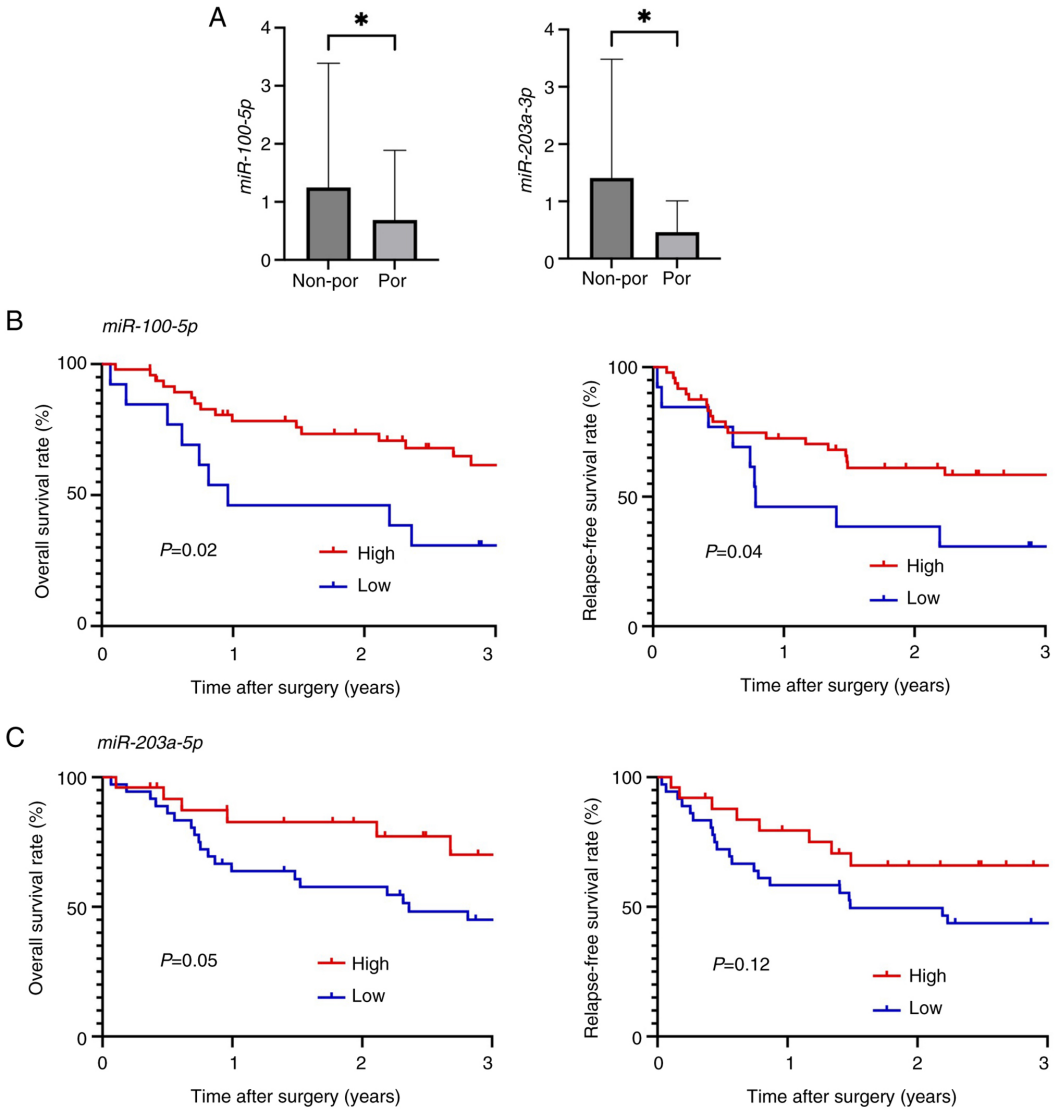
(A) Relationship between tumor differentiation and miR-100-5p and miR-203a-3p levels in clinical samples. (B and C) Kaplan-Meier curves for overall survival and relapse-free survival rates in patients stratified by (B) miR-100-5p levels and (C) miR-203a-3p levels. All the quantitative measurements were performed in triplicate, and data for relative quantity are presented as the mean ± SD. *P<0.05. por, poorly differentiated. non-por, well- to moderately differentiated.

Next, the clinical significance of miR-100-5p and miR-203a-3p was examined in patients with ESCC. The expression levels of miR-100-5p and miR-203a-3p were significantly lower in patients with por ESCC than in patients with non-por ESCC (P=0.02 and P=0.04, respectively) (Fig. 2A). Based on these results, a receiver operating characteristic curve was constructed according to histological differentiation and divided the expression levels of miR-100-5p and miR-203a-3p into high-expression and low-expression groups for further analysis.

In the low *miR-100-5p* group, tumor differentiation was poorer, and the depth of invasion was greater compared with the high miR-100-5p group (P<0.001 and P=0.012, respectively). Similarly, in the low miR-203a-3p group, tumor differentiation was poorer, the depth of invasion was greater, and lymph node metastasis occurred more frequently compared with the high miR-203a-3p group (P=0.01, P=0.008, and P=0.03, respectively) (Table I).

As demonstrated in Fig. 2B, OS and RFS rates were significantly worse in the low miR-100-5p group compared with the high miR-100-5p group (P=0.02 and P=0.04, respectively). Additionally, the OS rate was significantly worse in the low miR-203a-3p group than in the high miR-203a-3p group, while the RFS rate was not statistically significant (P=0.05 and P=0.12, respectively; Fig. 2C).

### Expression and function of miR-100-5p and miR-203a-3p in ESCC cell lines

**Figure 3.**
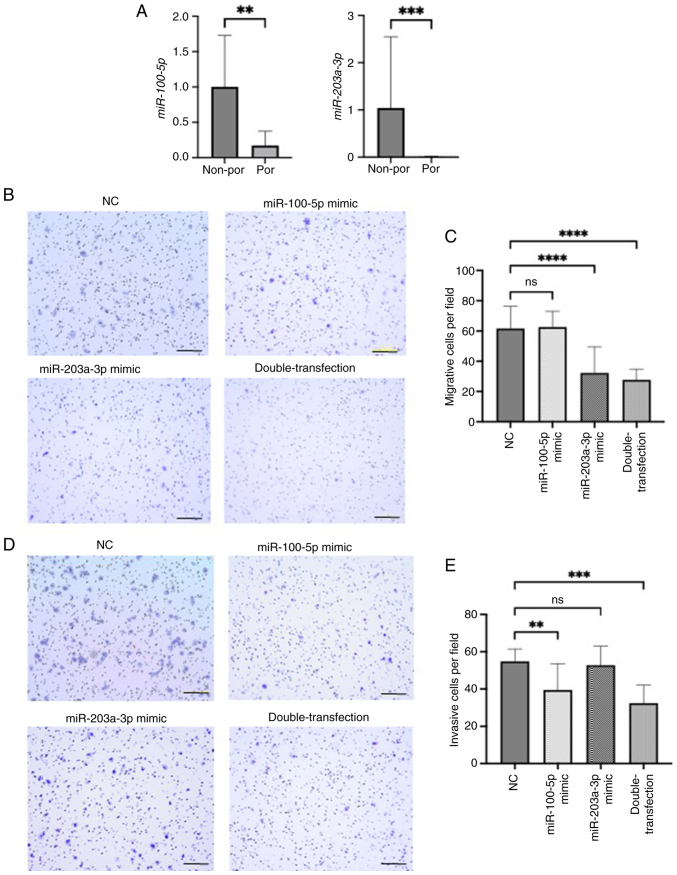
(A) Relationship between tumor differentiation and miR-100-5p and miR-203a-3p levels in non-por and por cell lines. Representative images and quantitative measurement of (B and C) migration and (D and E) invasion assays induced by miR-100-5p and miR-203a-3p mimics in KYSE70 cells. For double-transfection experiments, both miR-100-5p and miR-203a-3p mimics were co-transfected at 10 nM each. Scale bar, 200 µm. All the quantitative measurements were performed in triplicate, and data for relative quantity are presented as the mean ± SD. **P<0.01, ***P<0.001 and ****P<0.0001 compared with NC. miR, microRNA; por cell lines, poorly differentiated cell lines (KYSE70, KYSE110, TE5) non-por cell lines, well- to moderately differentiated cell lines (TE4, TE8, TE11). NC, negative control.

Furthermore, the function of miR-100-5p and miR-203a-3p was examined in ESCC cell lines. First, their expression levels were measured in ESCC cell lines and it was found that miR-100-5p and miR-203a-3p were significantly lower in poorly differentiated ESCC cell lines compared with well- and moderately differentiated cell lines (Fig. 3A). This expression pattern was consistent with our clinical findings that patients with high expression of these miRNAs had higher degree of differentiation and more favorable prognosis.

To investigate the functional roles of these miRNAs in ESCC progression, cell migration and invasion assays were conducted using miRNA mimics in KYSE70 cells. Transfection efficiency for each mimic is shown in Fig. S3A. Cell migration was significantly inhibited by the miR-203a-3p mimic alone and by the combination of miR-100-5p and miR-203a-3p mimics compared with the negative control mimic (Fig. 3B and C). This result indicates that miR-203a-3p plays a suppressive role in the migratory ability of ESCC cells. Similarly, cell invasion was significantly suppressed by the miR-100-5p mimic and by the double transfection, indicating that miR-100-5p mainly contributes to the inhibition of invasive potential (Fig. 3D and E). These results suggested that both miRNAs cooperatively suppress ESCC progression through inhibition of migration and invasion.

### Potential target of miR-100-5p and miR-203a-3p

**Figure 4.**
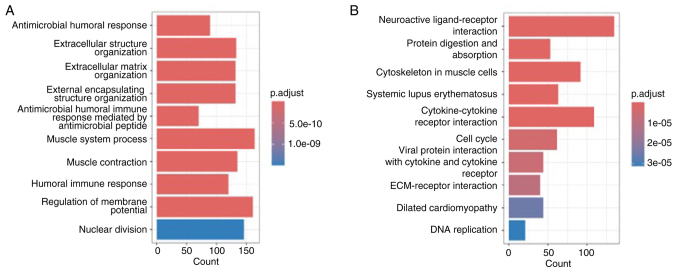
Biological functions of the *FKBP5* gene. (A) Gene Ontology enrichment analysis of the *FKBP5* gene. (B) Kyoto Encyclopedia of Genes and Genomes enrichment analysis of the *FKBP5* gene.

**Figure 5.**
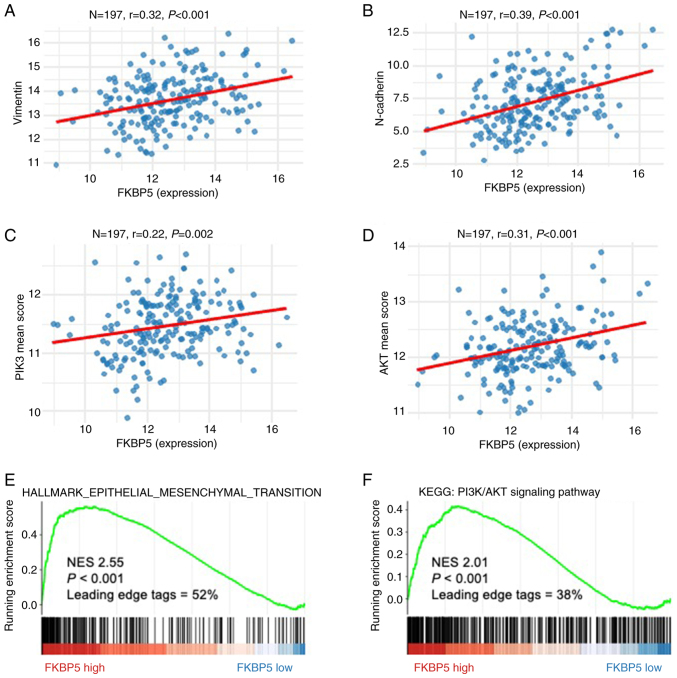
Biological functions of the *FKBP5* gene. (A-D) Positive association between *FKBP5* and (A) Vimentin, (B) N-cadherin, (C) PIK3 family and (D) AKT family. (E and F) *FKBP5* regulated (E) epithelial-mesenchymal transition and (F) PI3K/AKT signaling pathway using the Gene Set Enrichment Analysis.

*FKBP5* was identified as a putative downstream target of both miR-100-5p and miR-203a-3p using TargetScan Human 8.0 (Fig. S4). To clarify the functional relevance of *FKBP5* in ESCC, comprehensive bioinformatics analyses were first performed. GO and KEGG enrichment of *FKBP5*-associated genes revealed significant overrepresentation of pathways involved in cell cycle control, nuclear division and extracellular matrix organization (Fig. 4A and B). Moreover, Pearson correlation (Fig. 5A-D) and pre-ranked GSEA (Fig. 5E and F) demonstrated robust positive associations between *FKBP5* expression and both epithelial-mesenchymal transition (EMT) and PI3K/AKT signalling pathway in ESCC.

**Figure 6.**
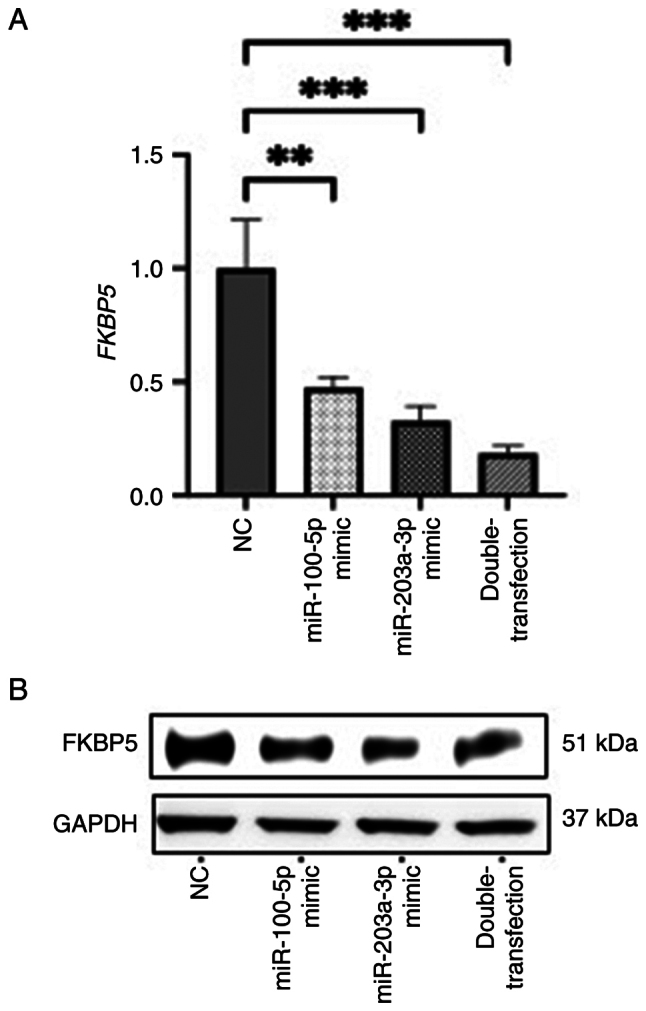
*FKBP5* expression in KYSE70 cells transfected with miR-100-5p and miR-203a-3p mimics. (A) Relative mRNA expression levels measured by reverse transcription-quantitative PCR. (B) Protein levels determined by western blotting. **P<0.01 and ***P<0.001. miR, microRNA; NC, negative control.

Experimentally, transfection of miR-100-5p and miR-203a-3p mimics significantly decreased both *FKBP5* transcript and protein levels, with the combined transfection of both miRNAs producing the most pronounced reduction (Fig. 6A and B). These results suggested that *FKBP5* is a downstream target of both miRNAs and that the two miRNAs may act cooperatively to suppress its expression. To further confirm the functional involvement of *FKBP5* in the miRNA-mediated suppression of ESCC cell motility, rescue experiments were performed using the double-transfection of miR-100-5p and miR-203a-3p mimics with or without *FKBP5* siRNA. Adding *FKBP5* siRNA to the double-transfection did not produce additional reductions in migration or invasion compared with the double-transfection alone, indicating that the effects of the mimics are largely mediated by *FKBP5* suppression (Fig. S5). In clinical ESCC samples, the expression levels of miR-100-5p/miR-203a-3p and *FKBP5* protein exhibited a negative correlation trend; however, this association did not reach statistical significance (Fig. S6).

**Figure 7.**
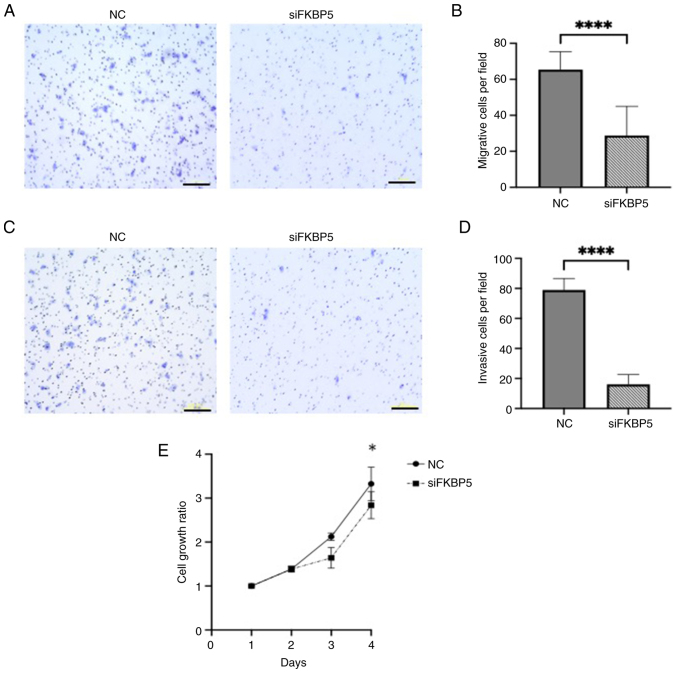
Representative images and quantitative measurement of (A and B) migration, (C and D) invasion and (E) cytotoxicity assays induced by *FKBP5* suppression in KYSE70 cells. Scale bar, 200 µm. All the quantitative measurements were performed in triplicate, and data for relative quantity are presented as the mean ± SD. *P<0.05 and ****P<0.0001 compared with NC. NC, negative control; si-, small interfering.

Functional assays further demonstrated that *FKBP5* knockdown, whose efficiency was confirmed by western blotting (Fig. S3B), significantly impaired ESCC cell migration, invasion, and increased cytotoxicity compared with negative controls (Fig. 7A-E), suggesting that *FKBP5* promotes malignant phenotypes in ESCC cells. Additionally, apoptosis assays were performed. Annexin V/PI flow cytometry showed no significant differences between *FKBP5* siRNA and controls (Fig. S7A). Western blotting revealed slight changes in Caspase-3/Cleaved Caspase-3 and a marked reduction in Bcl-2 (Fig. S7B). These results suggested that *FKBP5* knockdown alone did not provide definitive evidence for apoptosis involvement.

**Figure 8.**
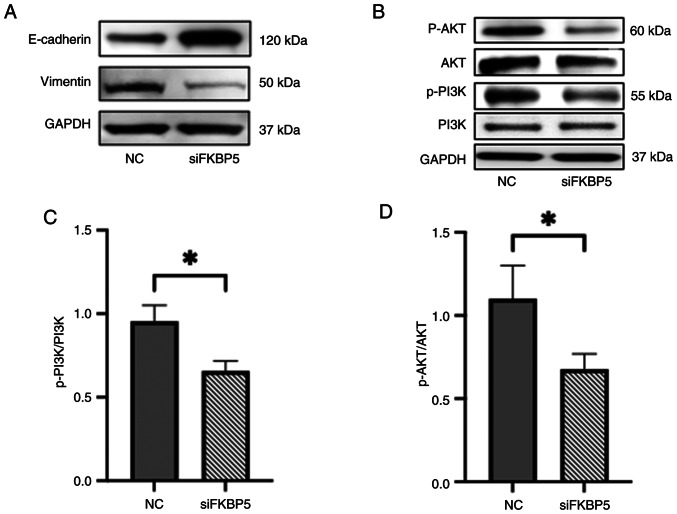
Characterization of (A) epithelial-to-mesenchymal transition markers (E-cadherin and Vimentin) and (B) PI3K/ AKT signaling pathway components, including p-PI3K, p-AKT and total (PI3K and AKT) proteins, in response to *FKBP5* regulation by western blotting in KYSE70 cells. Quantification of (C) p-PI3K/ PI3K and (D) p-AKT/ AKT ratios expressed as fold change relative to NC. *P<0.05. p-, phosphorylated; NC, negative control; si-, small interfering.

Furthermore, the effect of *FKBP5* on EMT and PI3K/AKT pathway activity was investigated by performing western blotting for key EMT markers and phosphorylated signaling proteins. *FKBP5* knockdown led to upregulation of the epithelial marker E-cadherin and downregulation of the mesenchymal marker vimentin (Fig. 8A). In addition, knockdown of *FKBP5* reduced the levels of p-PI3K and p-AKT, indicating suppression of the PI3K/AKT signalling pathway (Fig. 8B-D). These findings indicated that *FKBP5* facilitates EMT and activates the PI3K/AKT pathway in ESCC cells, and that its suppression may help reverse the mesenchymal phenotype and inhibit oncogenic signaling associated with invasion and metastasis.

### Prognostic impact of FKBP5 expression in patients with ESCC

**Figure 9.**
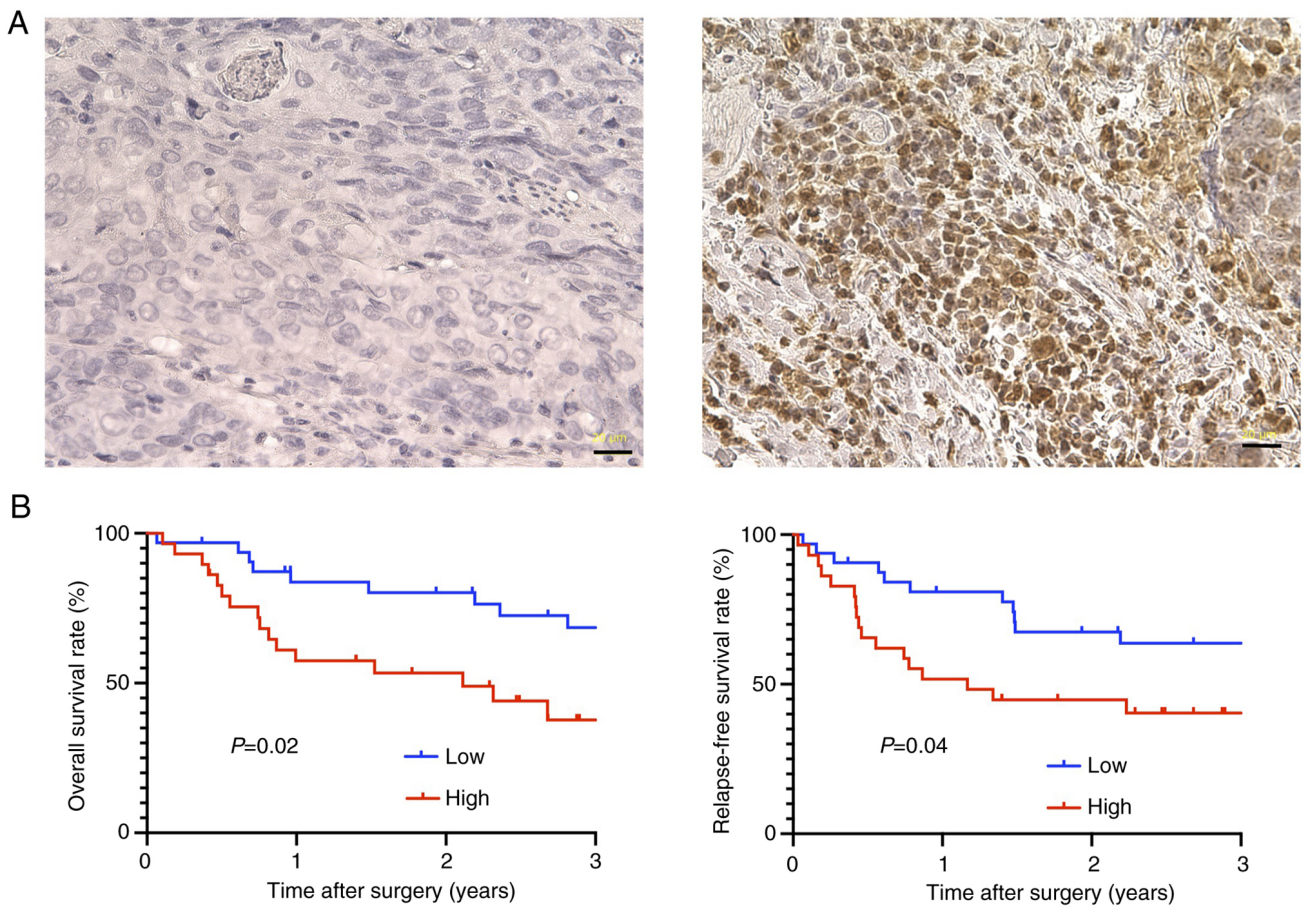
(A) Representative immunohistochemical staining for *FKBP5* in esophageal squamous cell carcinoma tissues by IHC. Scale bar, 20 µm. (B) Kaplan-Meier curves for overall survival and relapse-free survival rates in patients stratified by *FKBP5*.

Finally, the prognostic significance of *FKBP5* expression was evaluated in patients with ESCC using IHC. Representative IHC images are shown in Fig. 9A. Based on the median H-score, patients were categorized into high- and low-*FKBP5* expression groups. Survival analysis revealed that patients in the high *FKBP5* expression group exhibited significantly poorer OS and RFS compared with those in the low expression group (Fig. 9B). These findings suggested that elevated *FKBP5* expression is associated with adverse clinical outcomes in ESCC and may serve as a potential prognostic biomarker.

## Discussion

**Figure 10.**
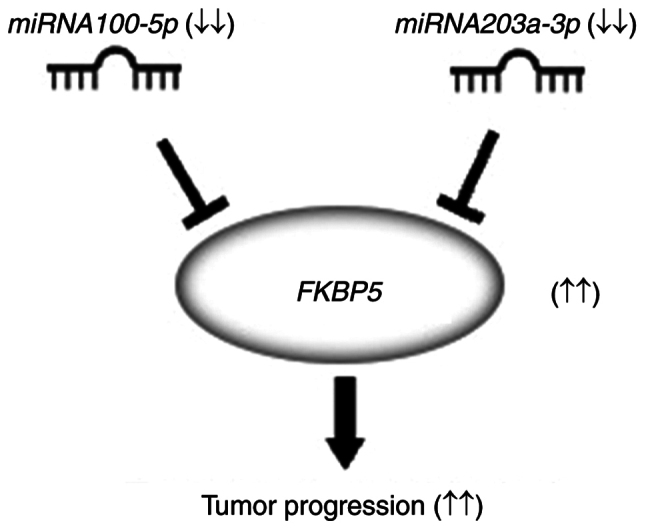
Schematic representation of the proposed role of miR-100-5p and miR-203a-3p in esophageal squamous cell carcinoma. miR-100-5p and miR-203a-3p inhibit tumor progression by targeting *FKBP5*. miRNA or miR, microRNA.

In the present study, it was aimed to elucidate the role of miRNAs in tumor characteristics within ESCC, specifically focusing on variations in tumor differentiation. Our findings strongly suggest that patients with elevated levels of miR-100-5p and miR-203a-3p have a favorable prognosis. Furthermore, it was established that these miRNAs inhibit cellular migration and invasion in ESCC by targeting *FKBP5*, a promising candidate for therapeutic intervention in ESCC (Fig. 10).

First, tumor differentiation in ESCC was investigated. Por ESCC exhibits distinct characteristics compared with non-por ESCC. Patients with por ESCC have higher rates of lymph node metastasis and recurrence. Furthermore, the pattern of lymph node metastasis in por ESCC has been reported to differ significantly from that in non-por ESCC (5).

Therefore, it was hypothesized that highly malignant por ESCC could provide insights into identifying molecules that play a crucial role in the tumor characteristics of ESCC. Through screening using a comprehensive miRNA array-based approach and public databases, two miRNAs were identified as potential candidates.

There are already studies on each of the candidate miRNAs. Regarding miR-100-5p, it suppresses CXCR7 expression, which is implicated in initiation, adhesion, angiogenesis and metastasis (13), and inhibits the activation of the PI3K/AKT signaling pathway, thereby suppressing lymphangiogenesis (14). Similarly, miR-203a-3p suppresses the activation of the PI3K/AKT signaling pathway, which is associated with tumor initiation and therapy resistance in ESCC (15).

From a clinical perspective, high expression levels of these miRNAs are linked to an improved prognosis in ESCC (16,17). The novelty of the present study lies in identifying that both miR-100-5p and miR-203a-3p are associated with tumor differentiation in ESCC using clinical data and cell lines. Furthermore, the combination of miR-100-5p and miR-203a-3p synergistically suppressed the malignant behavior of ESCC. In the present study, miR-100-5p alone did not sufficiently suppress cell migration, while miR-203a-3p alone was ineffective in suppressing cell invasion, possibly due to differences in transfection efficiency or concentration. Our intention was to demonstrate that simultaneous transfection of both miRNAs more effectively suppresses both migration and invasion.

*FKBP5* was identified as a potential target of the combined action of miR-100-5p and miR-203a-3p. *FKBP5* has previously been reported as an intracellular protein that promotes cancer progression and chemoresistance by activating the NF-κB signaling pathway (18–21). In the present study, western blot analysis and public database data clearly demonstrated that *FKBP5* also activates the PI3K/AKT signaling pathway, thereby contributing to enhanced malignancy in ESCC (22). Moreover, siRNA-mediated *FKBP5* knockdown significantly affected migration, invasion, cytotoxicity, and several signaling proteins, whereas no appreciable changes were detected in apoptosis assays using flow cytometry or western blotting. These findings suggested that the oncogenic function of *FKBP5* in ESCC may be more strongly associated with cell motility and survival signaling rather than direct regulation of apoptosis.

*FKBP5* is known to play an oncogenic role in various malignancies, including thyroid carcinoma, renal clear cell carcinoma and melanoma (23–25). In gastrointestinal cancers, similar oncogenic functions have been reported in esophageal adenocarcinoma, gastric cancer and colorectal cancer, where elevated *FKBP5* expression is associated with poor prognosis (26–28). Collectively, these findings suggested that *FKBP5* contributes to poor prognosis by promoting EMT and activation of the PI3K/AKT signaling pathway, which is consistent with our results. To the best of our knowledge, this is the first study to demonstrate that *FKBP5* promotes tumor aggressiveness through regulation by miR-100-5p and miR-203a-3p in ESCC.

Based on the results of the present study, *FKBP5* has the potential to become an important therapeutic target for ESCC in the future. Therefore, suppressing *FKBP5* through the administration of both miR-100-5p and miR-203a-3p may represent an effective treatment option for patients with ESCC. Further basic and clinical studies are necessary to evaluate the efficacy of this treatment strategy in suppressing *FKBP5* for ESCC.

The present study has several limitations. It was conducted at a single institution with a limited number of patients. In the GEO dataset used, miR-203a-3p was not listed, which precluded combined analyses of expression arrays and public databases. While these miRNAs appeared to be associated with tumor differentiation, it remains unclear whether they and *FKBP5* are directly involved in the differentiation process. Furthermore, dual-luciferase reporter assays were not performed due to the lack of necessary equipment for gene transfection in our institution. As a result, the direct interaction between these miRNAs and *FKBP5* was not validated. However, Chen *et al* (29) reported such an interaction. In our functional rescue experiment, adding si*FKBP5* to miRNAs mimic-treated cells did not alter the suppression of migration and invasion, consistent with the authors' hypothesis that *FKBP5* mediates these effects. Additionally, although a negative correlation between miR-100-5p/miR-203a-3p and *FKBP5* expression was observed, the association did not reach statistical significance (Fig. S6). However, it is considered that a larger cohort study may confirm a statistically significant negative correlation. Moreover, cell viability was assessed using the CCK-8 assay, which measures metabolic activity as an indirect indicator of viable cell number; therefore, it may not directly reflect cell proliferation rates. Additional assays based on DNA synthesis or proliferation markers would provide a more definitive evaluation of proliferation. Nevertheless, our primary aim was to identify miRNAs associated with tumor characteristics and potential therapeutic targets in ESCC, and this objective was achieved. In conclusion, miR-100-5p and miR-203a-3p inhibited tumor progression by targeting *FKBP5* in ESCC, highlighting *FKBP5* as a promising therapeutic target in ESCC.

## Supplementary Material

Supporting Data

## Figures and Tables

**Table I.** Clinicopathological characteristics of patients.

	Expression level of miR-100-5p			Expression level of miR-203a-3p		
		
Characteristics	Low (n=13)	High (n=48)	P-value	Low (n=36)	High (n=25)	P-value
Age (years)^a^	72 (43–83)	78 (60–83)	0.12	74 (43–83)	74 (57–83)	0.65
Sex^b^						
Male	12 (92.3)	36 (75.0)	0.26	29 (80.5)	19 (76.0)	0.75
Female	1 (7.7)	12 (25.0)		7 (19.5)	6 (24.0)	
Tumor differentiation^b^						
Well	0 (0)	18 (37.5)	<0.001	14 (38.9)	4 (16.0)	0.01
Moderately	2 (15.4)	21 (43.8)		8 (22.2)	15 (60.0)	
Poorly	11 (84.6)	9 (18.7)		14 (38.9)	6 (24.0)	
Pathological T stage^b^						
T1	0 (0)	17 (35.4)	0.012	5 (13.9)	12 (48.0)	0.008
T2, T3, T4	13 (100)	31 (64.6)		31 (86.1)	13 (52.0)	
Pathological N stage^b^						
N0	3 (23.1)	23 (48.0)	0.13	11 (30.6)	15 (60.0)	0.03
N1, N2, N3	10 (76.9)	25 (52.0)		25 (69.4)	10 (40.0)	
Lymphatic invasion^b^						
ly0	4 (30.8)	23 (47.9)	0.35	13 (36.1)	14 (56.0)	0.19
ly1, ly2, ly3	9 (69.2)	25 (52.1)		23 (63.9)	11 (44.0)	
Vascular invasion^b^						
v0	3 (23.1)	14 (29.2)	1.00	8 (22.2)	9 (36.0)	0.26
v1, ν2, ν3	10 (76.9)	34 (70.8)		28 (77.8)	16 (64.0)	

Data are expressed as number (%) or median (range).

a Mann-Whitney U test;

b Fisher's exact test. miR, microRNA.

## Data Availability

The data generated in the present study may be found in the Gene Expression Omnibus under accession number GSE43732 or at the following URL: https://www.ncbi.nlm.nih.gov/geo/query/acc.cgi?acc=GSE43732.

## Abbreviations

por: poorly differentiated carcinoma
non-por: well- to moderately differentiated carcinoma
ESCC: esophageal squamous cell carcinoma
miRNA or miR: microRNA
