# Supplementary material for: miR-100-5p and miR-203a-3p suppress esophageal squamous cell carcinoma progression by targeting FKBP5
Source: Oncol Rep. 2025 Oct 3;54(6):170. doi: 10.3892/or.2025.9003 (PMC12521887; doi:10.3892/or.2025.9003)

Figure S1. Flow diagram illustrating the inclusion and exclusion criteria for the selection of patients with esophageal squamous cell carcinoma. The numbers of patients excluded at each stage and the final number included in the analysis are indicated.

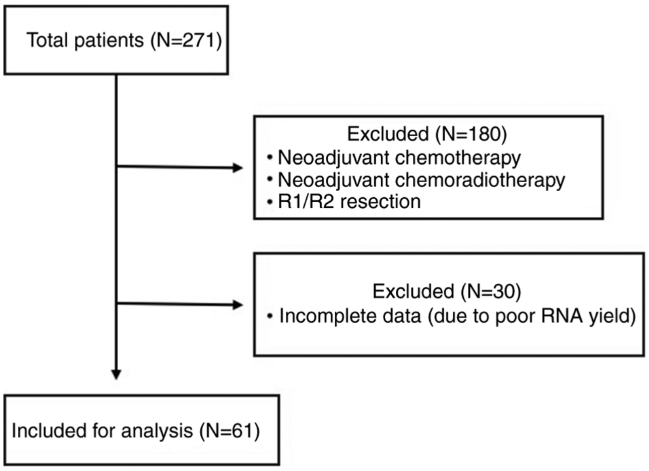

Figure S2. Kaplan-Meier curves for overall survival in patients stratified by tumor differentiation status, based on Gene Expression Omnibus dataset analysis. The analysis included 119 patients with esophageal squamous cell carcinoma who underwent surgery alone without receiving chemotherapy and radiotherapy, n=119 (por: n=32, non-por: n=87). por, poorly differentiated; non-por, well- to moderately differentiated.

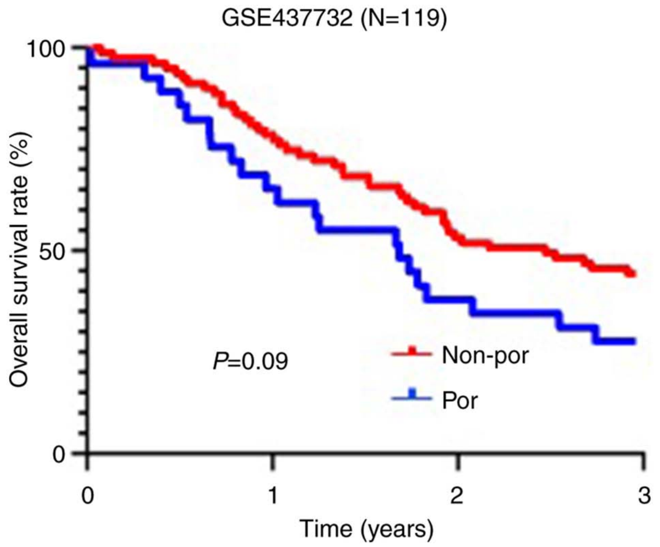

Figure S3. (A) Transfection efficiency of miR-100-5p and miR-203a-3p mimics in KYSE70 cells, as measured by relative miRNA expression levels using reverse transcription-quantitative PCR compared with NC. (B) Knockdown efficiency of si*FKBP5* was confirmed by western blotting. miRNA or miR, microRNA; NC, negative control; si-, small interfering.

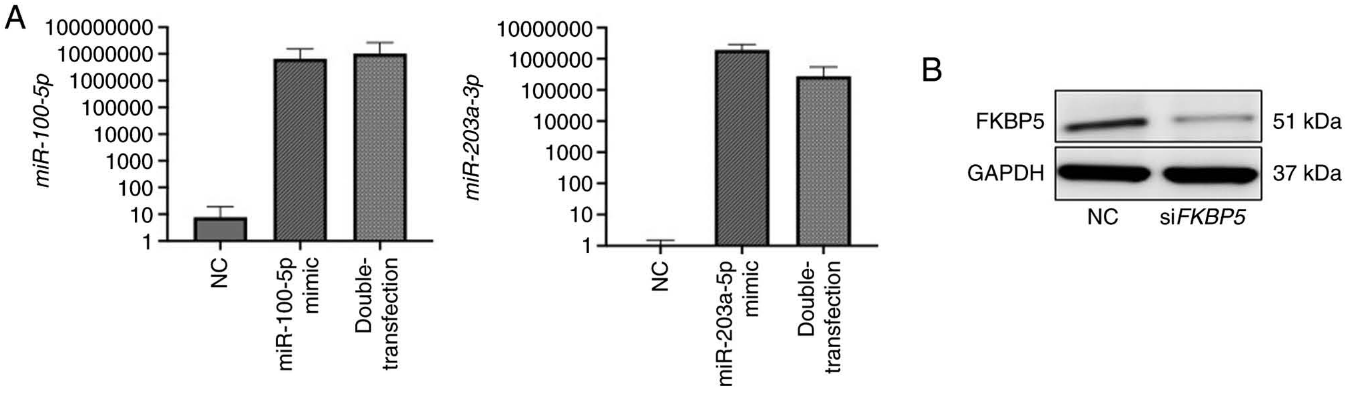

Figure S4. Putative sequences of miR-100-5p and miR-203a-3p within *FKBP5* mRNA with the binding sites. miR, microRNA.

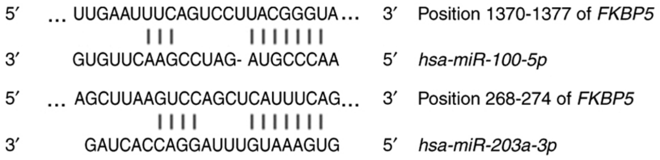

Figure S5. Representative images and quantitative measurement of (A and B) migration and (C and D) invasion assays in KYSE70 cells treated with double-transfection alone or in combination with si*FKBP5*. For double-transfection experiments, both miR-100-5p and miR-203a-3p mimics were co-transfected at 10 nM each. Scale bar, 200 μm. All the quantitative measurements were performed in triplicate, and data for relative quantity are presented as the mean ± SD. miR, microRNA; si-, small interfering.

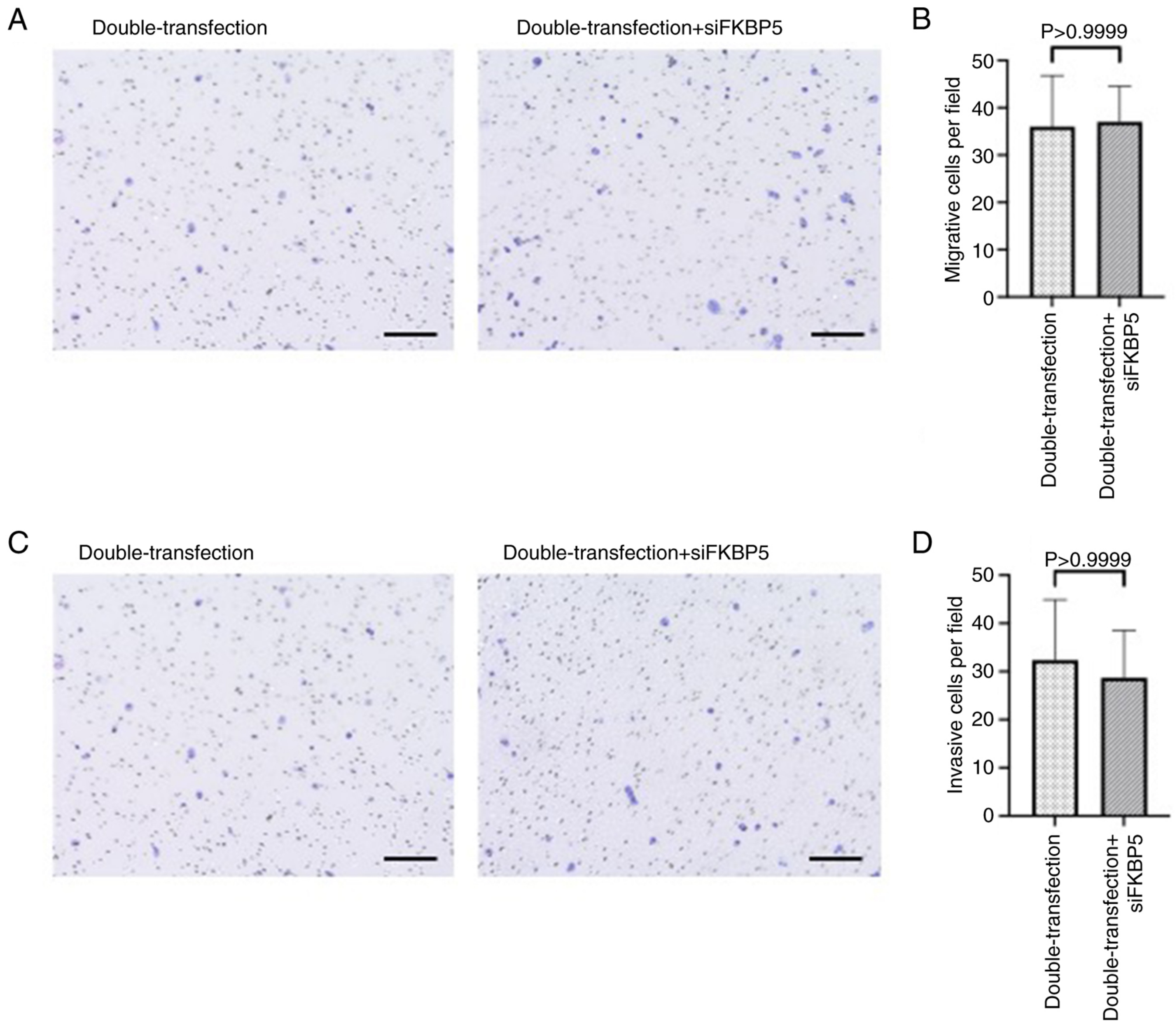

Figure S6. Relationship between miR-100-5p/miR-203a-3p and *FKBP5* protein levels in clinical specimens. miR, microRNA.

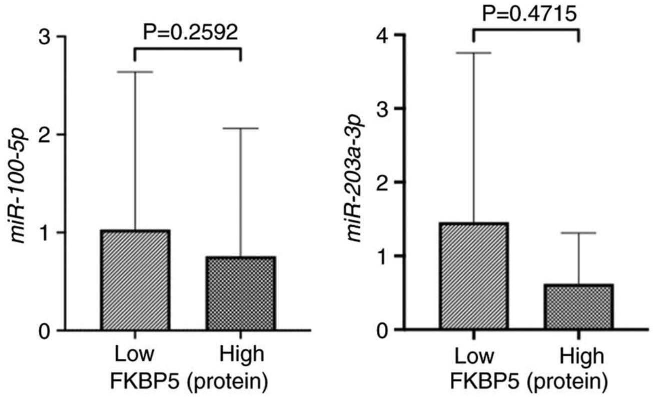

Figure S7. Apoptosis assays following *FKBP5* knockdown in KYSE70 cells. (A) Flow cytometry of Annexin V/PI staining in NC and si*FKBP5* groups. (B) Western blot of apoptosis-related proteins (Caspase-3, Cleaved-Caspase-3 and Bcl-2) in NC and si*FKBP5* groups. NC, negative control; si-, small interfering.

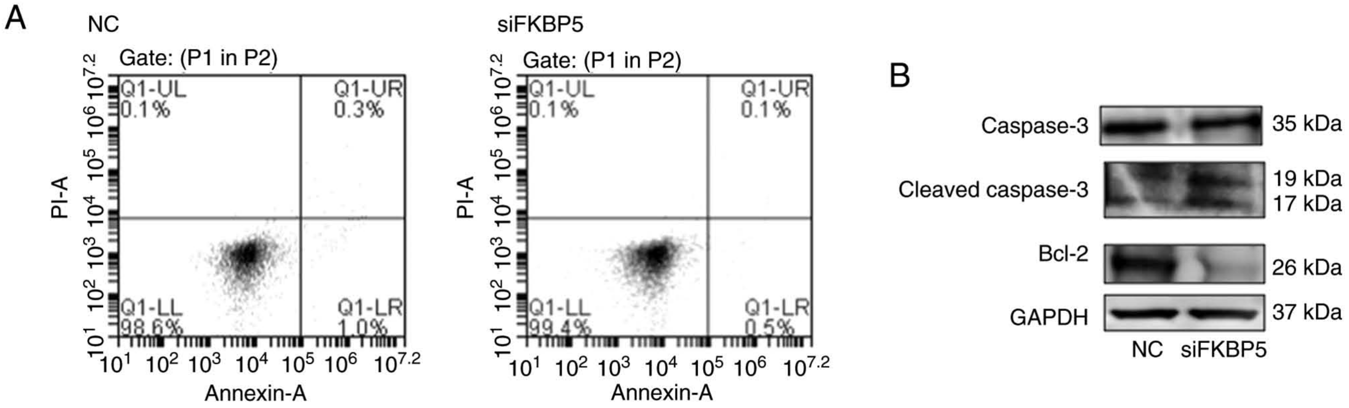

Supplement: Supporting Data [file Supplementary_Data.pdf]
